# Obesity Induced by Neonatal Overfeeding Worsens Airway Hyperresponsiveness and Inflammation

**DOI:** 10.1371/journal.pone.0047013

**Published:** 2012-10-08

**Authors:** Zehui Ye, Ying Huang, Dan Liu, Xiaoyi Chen, Dongjuan Wang, Daochao Huang, Li Zhao, Xiaoqiu Xiao

**Affiliations:** 1 Ministry of Education Key Laboratory of Child Development and Disorders, Chongqing Medical University, Chongqing, China; 2 Department of Respiratory Medicine, Chongqing Medical University, Chongqing, China; 3 Chongqing Key Laboratory of Translational Medical Research in Cognitive Development and Learning and Memory Disorders, Chongqing Medical University, Chongqing, China; 4 Key Laboratory of Pediatrics in Chongqing, Chongqing Medical University, Chongqing, China; 5 Chongqing International Science and Technology Cooperation Center for Child Development and Disorders, Children’s Hospital, Chongqing Medical University, Chongqing, China; University of Rochester Medical Center, United States of America

## Abstract

**Background:**

Obesity is a risk factor for the development of certain respiratory diseases, and neonatal overfeeding results in an early onset of obesity in adulthood. However, the influence of neonatal overfeeding on respiratory diseases has rarely been studied. Therefore, this paper is aimed at investigating the effect of neonatal overfeeding on airway responsiveness and inflammation.

**Methodology/Principal Findings:**

The neonatal overfeeding was induced by reducing litter size to three pups per litter (small litter, SL) in contrast to the normal litter size with ten pups per litter (NL) on postnatal day 3 (P3) in male ICR mice. On P21, mice were weaned to standard chow diet. Airway responsiveness to methacholine was measured either on P21 or P150. Total and classified inflammatory cells in bronchoalveolar lavage fluid (BALF) were counted, lung inflammatory cells were evaluated through staining with hematoxylin & eosin and F4/80 immunohistochemistry; lung fibrosis was evaluated through staining with Masson and α-SAM immunohistochemistry. Leptin levels in serum were measured by RIA; TNF-α levels in serum and BALF were quantified by ELISA; mRNA levels of TNF-α, CTGF and TGF-β1 in lung tissues were measured using real-time PCR. Mice from SL exhibited accelerated body weight gain, impaired glucose tolerance and hyperleptinemia. Enhanced airway responsiveness to methacholine was observed in SL mice on P150, but not on P21. Pulmonary inflammation was evident in SL mice on P150, as reflected by inflammatory cells especially macrophages around bronchi and interstitium. BALF and serum TNF-α levels and lung TNF-α mRNA expression were significantly increased in SL mice on P150. More collagen accumulated surrounding the bronchi on P150; lung mRNA levels of TGF-β1 and CTGF were also increased on P150.

**Conclusion:**

In addition to inducing a variety of metabolic defects, neonatal overfeeding enhanced lung inflammation, which may lead to airway remodeling and airway hyperresponsiveness in adulthood.

## Introduction

The rapid raise of obesity prevalence has already constituted a significant health and economic burden on our society as it is a major contributor to a variety of chronic diseases such as cardiovascular diseases, metabolic diseases, osteoarthritis, and cancers. However, the effects of obesity on the respiratory system are underappreciated. Since the pioneering research studied by Carmargo and his colleagues showing a parallel increase in the prevalence of obesity and asthma, a growing body of epidemiological evidence indicates an increased incidence of asthma in the overweight and obese population [1]–[5]. There are clear effects of obesity on pulmonary function, linked to enormous respiratory diseases such as asthma, chronic obstructive pulmonary disease, sleep apnea and so on. For instance, several studies reported that BMI was associated with an increased risk of developing airway hyperresponsiveness (AHR), an objective marker for asthma [6], [7]. In addition, obesity appears to increase asthma severity and influence the response to asthma controller medications [8], [9]. However, even though a possible causal association was established between obesity and asthma, the underlying mechanic basis is largely unclear.

The influence of obesity on asthma onset involves in a mechanical constraint on respiratory tracts, airway inflammation and organ remodeling. Obesity can alter respiratory function through affecting the thorax, diaphragm and abdominal muscles, which can increase impairment of the gas transport system [10]. Of particular importance is the role of airway inflammation in the development of AHR and asthma. For instance, several studies reported that asthmatic patients have elevated serum immunoglobulin E (IgE) antibody levels [11], increased production of pro-inflammatory cytokines such as tumor necrosis factor-α (TNF-α), and interleukin (IL)-8, IL-4 and IL-13, and significant recruitment of inflammatory cells into bronchoalveolar lavage fluid (BALF) and lung tissue [12]. The obese state has been characterized to create systemic low-grade inflammation as indicated by increased inflammatory markers [13]. TNF-α is an important mediators of the inflammatory response in obesity and is highly expressed in infiltrating macrophages and adipocytes,which may also play a role in AHR [12]–[14]. Chronic airway inflammation may lead to airway remodeling, another central feature of asthma [15]. Features of this remodeling process include epithelial shedding and subsequently results in the release of additional inflammatory cytokines, growth factors, and myofibroblast proliferation, smooth muscle hyperplasia and hypertrophy, and inflammatory cell infiltration. Two secreted factors, transforming growth factor β1 (TGF-β1) and connective tissue growth factor (CTGF), are widely regarded as universal mediators of fibrosis and organ remodeling. TGF-β1 has long been regarded as the most potent stimulator of collagen synthesis during lung fibrosis [16], [17]. Cell-based studies have shown that CTGF regulates multiple processes that contribute to lung fibrosis, and data from animal models of human disease also reported the importance of CTGF in fibrosis [18]. However, the detailed correlation between obesity, airway inflammation and remodeling remains to be elucidated.

The mechanistic basis of obesity and asthma has been thoroughly investigated by Shore and her colleagues through using several different mice models of obesity [19]–[23]. In these studies, either genetic ob/ob, db/db, carboxypeptidase E-deficient (Cpefat) mice or high fat diet induced obese mice exhibited innate AHR. Although ob/ob and db/db mice are extensively used for studies of obesity-related pathophysiology, mutations in the leptin gene (ob/ob) or its receptor (db/db) are rarely described in humans. In fact, high-fat diet induced obesity better resembles the development of human obesity. However, high-fat diet alters pulmonary responses to allergen [24], which makes it hard to distinguish the individual effects of obesity or high-fat diet while in study of increased asthmatic susceptibility in obese mice. Therefore, it is necessary to determine whether AHR occurs in other mice models of obesity. Previous studies showed that early neonatal overfeeding has significant impacts on the long-term regulation of body weight and contributes to the development of obesity in adulthood. One well-established model to study the effect of neonatal overfeeding is the manipulation of the size of mice litters at the early stage of life. When pups are raised in small litter, e.g. 3 pups/litter presumably milk intake in each individual pup is greater than its control that is raised in a normal sized litter with around 10 pups/litter. These chronic neonatal overfeeding mice are characterized by persistent overweight and early onset of obesity, hyperleptinemia, hyperinsulinemia, glucose intolerance, impaired hypothalamic feeding circuitry, impaired norepinephrine turnover and brown adipose tissue thermogenesis [25]–[28]. In the present study, we investigate the short-term and long-term effects of neonatal overfeeding on pulmonary function and inflammation.

## Materials and Methods

### Ethics Statement

All animal studies were performed in accordance with the Guide for the Care and Use of Laboratory Animals of the National Institutes of Health. All animals care and experimental protocols were approved by the Ethical Principles in Animal Research adopted by the Chongqing Medical University for Animal Experimentation.

### Animals and Protocols

Male offspring from ICR (Jackson Laboratories, Vital River, Beijing) pregnant mice were used in our study. Since consistent maternal care is critical for the outcome of this study, we chose the ICR mouse strain which is recognized for its excellent nurturing abilities and care of offspring. All animals were maintained under a 12 hr light/12 hr dark (lights on at 0700 h) cycle and constant temperature (23±2°C). Pregnant ICR mice were maintained on standard chow diets (containing 10% fat, 70% carbohydrates and 20% protein by energy, supplied by Shanghai Laboratory Animal Center (SLAC), Chinese Academy of Sciences, Shanghai, China), housed individually and monitored closely for the day of birth, which was considered as postnatal day 0 (P0). Neonatal overfeeding was induced by reducing litter size to 3 pups per litter (small litter, SL) on P3, while normal litters (NL) were culled to 10 pups per litter and nurtured by their own mother as control. The male pups were weaned onto standard chow diet on P21 and housed 3 per cage. Food and water were available *ad libitum* unless fasting was required for the experiment. These mice were sacrificed by decapitation on P21 and P150 between 10∶00 and 12∶00 AM. Each group contained 10–15 mice.

### Glucose Tolerance Test (GTT)

GTT was performed on P120. After 16 hours of fasting, the mice from NL and SL received a 20% glucose solution (2 g/kg) through intraperitoneal injection. Blood glucose concentration was measured from the tail vein immediately at 0, 15, 30, 60 and 120 minutes after glucose loading. Blood glucose levels were measured using a glucometer (SureStep OneTouch, Amecira) [29]. Area under the curve (AUC) measurement across 120 min was determined from the average for each animal, using the trapezoidal method with baseline set as the blood glucose levels at 0 min.

### Determination of Airway Hyperresponsiveness

Airway hyperresponsiveness was examined on P21 and P150. The tested mice were put into the whole-body plethysmograph (EMKA Technologies, Paris, France). Mice were exposed to aerosolized saline (for the baseline measurement) and increasing concentrations of methacholine (3.125, 6.25, 12.5, 25, 50 mg/ml) for 3 min each. Data were recorded and averaged for 5 min after 2 min rest. The index of airflow obstruction was expressed as enhanced pause (Penh, dimensionless parameter), which correlates with pulmonary airflow resistance. Penh is a dimensionless value that represents a function of the ratio of peak expiratory flow (PEF) to peak inspiratory flow (PIF) and a function of the timing of expiration (Pause) (Penh = PEP/PIF×Pause). Penh was calculated based on the EMKA Datanalyst provided by EMKA Technologies.

### BALF Assays

Mice were anesthetized with 10% chloral hydrate, exsanguinated and then sacrificed. The trachea was cannulated and bronchoalveolar lavage fluid (BALF) was collected by three injections of 0.5 ml phosphate-buffered saline (PBS) into lungs. Total BALF cells were collected by centrifugation, treated with red blood lysis buffer to remove the red blood cells and counted by microscopy using cell counter. Classified cells were performed with Wright–Giemsa, and then counted on a total of 200 cells under immersion oil at×1,000 magnification. The remaining lavage fluid was centrifuged at 1500 r/min for 10 min and the collected supernatant was analyzed for cytokine TNF-α.

### Histological Analysis

The left lungs were fixed in 4% paraformaldehyde at least for 72 h,dehydrated in graded alcohol series, cleared with dimethylbenzene, and embedded in paraffin. Serial sections of 5 µm thickness were stained with hematoxylin-eosin (H&E). Lung fibrosis was evaluated by Masson staining for collagen accumulation according to the manufacture’s protocols. Bronchi and lung alveoli were evaluated under a Nikon Eclipse E200 microscope adapted to a Nikon Coolpix 995 camera. Total inflammatory cell counts were determined from these images at 400×magnification in each slide, using the Imagelab Analysis software, and expressed as number of cells mm^−2^. In each mouse, three different areas were counted, and then the mean values were calculated. Such quantification was focused on peri-bronchiolar areas and alveolar septum, and these regions were the main sites of inflammatory reaction.

For immunohistochemical analysis of macrophages, lung sections were rinsed with phosphate-buffered saline (PBS 0.01 M), put into sodium citrate for antigen retrieval, cooled down for 5 min after boiling, and then incubated in 3% hydrogen peroxide for 10 min. After three washes with PBS, the sections were placed in PBS supplemented with 3% bovine serum albumin for 30 minutes. Anti-mouse antibody F4/80 (Abcam, ab6640, Hong Kong) or α-SAM (Abcam, ab5694, Hong Kong) was incubated overnight at 4°C in a dilution of 1∶200, a negative control was incubated by PBS instead of antibody. After washing with PBS, the sections were exposed to biotinylated universal secondary antibodies for 1 hour, then to streptavidin biotin horseradish peroxidase solution. The reaction product was developed using 3,3′-Diaminobenzidine tetrahydrochloride (Sigma). Sections were counterstained with hematoxylin for 20 s, dehydrated through graded alcohols, and mounted in resinous medium.

### Cytokine Analysis

Concentrations of TNF-α in BALF and serum were measured by Enzyme-Linked Immunoassays using a commercially available kit (CSB-E04741m, Cusabio biotech, Wuhan).

### Quantitative Real-time PCR

The frozen lung samples were homogenized in Trizol reagent (Takara Bio, Dalian, China), and RNA purification was performed by Trizol protocol. First-strand complimentary DNA (cDNA) was synthesized from total RNA according to the RNA PCR kit (Promega, Madison, WI), following the manufacturer’s protocol. Reverse transcription was performed using 1 µg of total RNA in 5.9 µl of the following solution: 2.0 µl 10×RT buffer, 2 µl 10×RT Random Primer, 0.8 µl 25×dNTP, 1.0 µl Multiscribe Reverse Transcriptase, 0.1 µl RNase inhibitor, and the addition of nuclear-free water to final volume of 20 µl. Reaction system was run at 25°C for 10 min, 37°C for 120 min and 85°C for 5 min. Primers for TNF-α (NM_013693.2), connective tissue growth factor (CTGF, NM_010217.2), transforming growth factor β1 (TGF-β1, NM_011577.1) and β-actin (NM_007393.3) were designed using Primer Express software from Applied Biosystems and the sequences are available upon request. PCR reactions were carried out in a 25 µl reaction buffer that included 12.5 µl SYBR GREEN, 0.25 µl of forward primer, 0.25 µl of reverse primer, 2 µl of cDNA, and 10 µl ddH2O and performed in triplicate for each sample in the BIO-RAD CFX96 Real-Time PCR system. The fluorescence intensity of each sample was measured at each temperature change to monitor amplification of the target gene. The comparative cycle time (CT) method was used to determine fold differences between samples. The comparative CT method determined the amount of target normalized to an endogenous reference (β-actin) and relative to a calibrator.

### Leptin Assays

A radioimmunoassay (RIA) kit (Linco, St. Charles, MO) was used to measure the serum levels of leptin [28]. The lowest detectable levels and intraassay variability were 0.25 ng/ml and 4.6%, respectively.

### Statistical Analysis

Data were expressed as the mean ± S.E.M. GraphPad Prism 4.0 program (San Diego, CA, USA) was used for statistical analysis. Student t-tests were used to determine significant differences among groups, with significance was defined as *P*<0.05.

## Results

### Neonatal Overfeeding Increases Body Weight Gain and Impairs Glucose Tolerance

**Figure 1:**
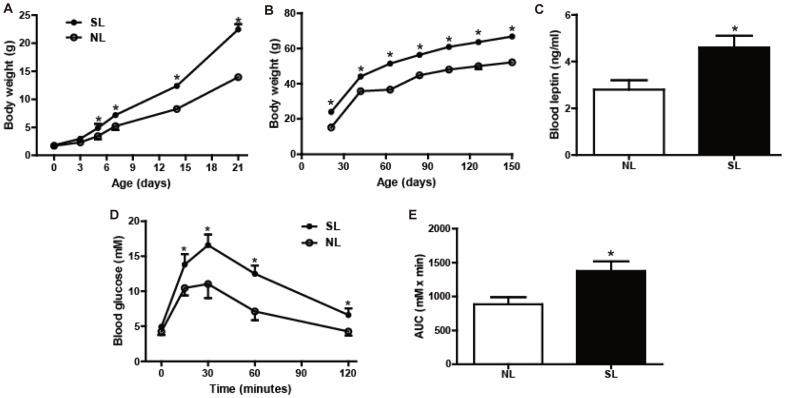
Neonatal overfeeding induces early-onset obesity and glucose intolerance in adulthood. Body weight was measured in normal litters (NL) (○; n = 12) and small litters (SL) (•; n = 12) groups during lactation (A) and after weaning (B); Blood leptin was measured on P150 using RIA (C). Intraperitoneal glucose tolerance test (GTT, D) was performed on P120, and blood glucose levels were evaluated at 0 (fasting), 15, 30, 60 and 120 minutes after glucose loading of NL(○, n = 6) and SL(•, n = 6), corresponding AUC from 0 to 120 min, with the glucose level at 0 min considered as the baseline (E). Data were expressed as mean± SEM, and the significant difference between two groups was analyzed by Student t-tests, *P<0.05.

During the period of lactation, mice from SL group displayed significant higher body weight from P7 (increased by 38%, *P*<0.05; Figure 1A) compared to NL group, reaching the highest difference on P21 (increased by 61%, *P*<0.05; Figure 1A). The higher body weight in SL mice were persistent until P150 (*P*<0.05; Figure 1B). Serum leptin levels of SL mice (4.6±0.5) were 1.6-fold higher than NL mice (2.8±0.4) on P150 (*P*<0.05; Figure 1 C).

On P120, fasting glucose levels were not significantly different between the SL and NL mice (4.9±0.8 and 4.2±0.5 mM). However, the GTT showed that blood glucose levels significantly increased at 15, 30, 60 and 120 min and AUC after glucose loading in SL mice compared with NL mice (Figure 1D and 1E).

### Neonatal Overfeeding Enhances Airway Responsiveness

**Figure 2:**
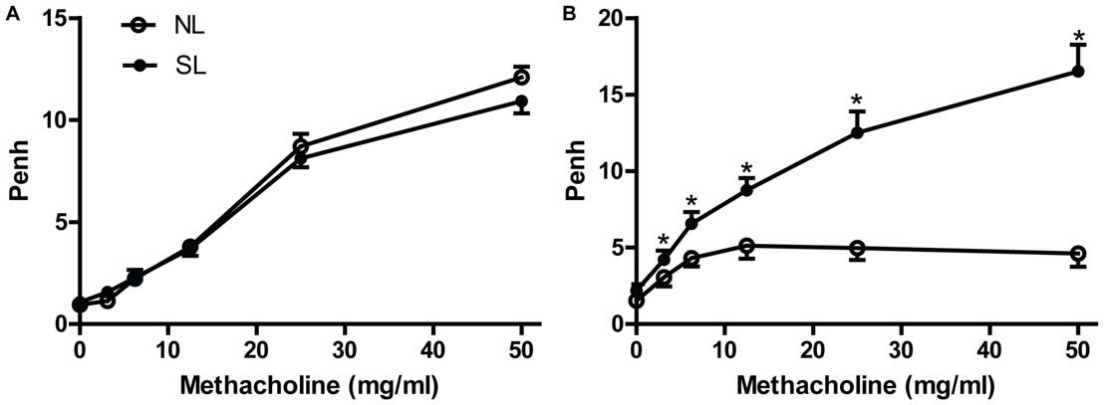
Airway responsiveness in response to increased doses of aerosolized methacholine was assessed in NL(○; n = 12) and SL(•; n = 12) groups at the age of 21-days (A) and 150-days (B). Readings were taken and averaged for 5 min after aerosolizing normal saline and increasing concentrations of methacholine (3.125, 6.25, 12.5, 25, 50 mg/ml). Data were expressed as mean± SEM, and the significant difference between two groups was analyzed by Student t-tests, *P<0.05.

Airway responsiveness was tested when the mice were on P21 (Figure 2A) and P150 (Figure 2B). There was no significant difference in baseline measurement between NL and SL mice on both P21 and P150. After aerosolizing methacholine, the average of Penh increased gradually with the increasing concentrations of methacholine (3.125, 6.25, 12.5, 25, 50 mg/ml), and the Penh of SL group was significant higher than NL group (*P*<0.05) on P150, which was not observed on P21 (Figure 2).

### Neonatal Overfeeding Increases Recruitment of Inflammatory Cells into Airway

**Figure 3:**
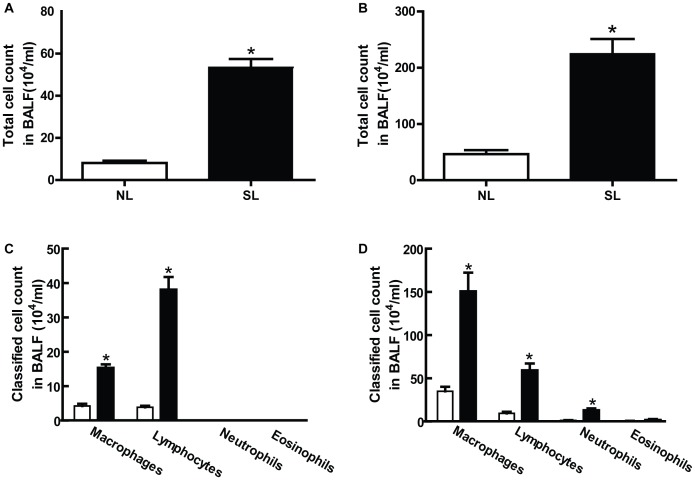
The effect of neonatal overfeeding on the total and classified cells number in BALF on P21 and P150. The total cells were counted with cell counter on P21 (A) and P150 (B); the classified cells were performed with Wright–Giemsa on P21 (C) and P150 (D). Data were expressed as mean± SEM, and the significant difference between two groups was analyzed by Student t-tests, *P<0.05.

The cell number was counted in BALF from both SL and NL mice on P21 and P150, and the data were shown in Figure 3. Generally, total cell number in BALF from P150 mice was higher than P21 mice in both SL and NL groups. Of note, the total cell number in BALF from the SL group was 6 and 5-fold higher than the NL group on P21 (Figure 3A) and P150 (Figure 3B), respectively. The number of macrophages and lymphocytes in BALF from SL mice was significantly higher than NL mice at the two tested time points. More neutrophils were observed in the SL group on P150, but not on P21. In addition, we observed very few eosinophils in BALF from SL mice on P150, which was almost totally undetected on P21 (Figure 3C and 3D).

**Figure 4:**
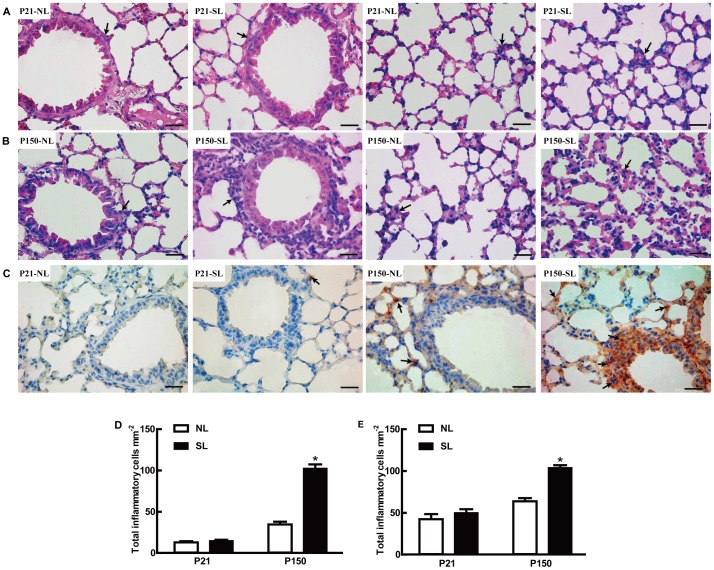
Neonatal overfeeding induces lung inflammatory cells infiltrating on P150. Mice lung tissue sections were stained with hematoxylin and eosin (H&E) for inflammatory cells on P21 (A) and P150 (B); the lung tissue sections were stained by F4/80 antibody for macrophage infiltration on P21 and P150 (C). Total inflammatory cells in peri-bronchiolar areas (D) and alveolar septum (E) were counted. Photomicrographs were taken at 400×magnification in each slide; scale bars  = 100 µm. The values of total inflammatory cells were averaged from 6 different mice in each group and expressed as mean±SEM. The significant difference between two groups was analyzed by Student t-tests, *P<0.05.

We then further confirmed increased airway inflammation in neonatal mice via lung histological analysis. Lung tissues from NL and SL mice on both P21 and P150 were examined with H&E staining. On P21, there was no significant difference of lung inflammation either in peri-bronchiolar areas or alveolar septum in both SL and NL mice (Figure 4A); On P150, H&E staining showed that infiltrated inflammatory cells in peri-bronchiolar areas (Figure 4B, left two panels) and alveolar septum (Figure 4B, right two panels) were significantly increased in SL mice compared to NL mice. The total inflammatory cells of SL mice were markedly higher in both peri-bronchiolar areas (about 3.0-fold, Figure 4D) and alveolar septum (about 1.6-fold, Figure 4E) compared with the NL groups on P150, but did not differ on P21. Lung inflammation was further defined by the presence of increased infiltration of inflammatory cells, which were examined with macrophage maker F4/80 antibody staining. The majority of infiltrated macrophages were found in peri-bronchiolar regions in SL mice on P150 (Figure 4C, right two panels), which were not observed in the lungs from P21 mice (Figure 4C, left two panels). We could hardly find any difference in macrophage accumulation around alveolar septum between the two groups at both ages (data not shown).

### Neonatal Overfeeding Increases Expression and Secretion of Pro-inflammatory Cytokines

**Figure 5:**
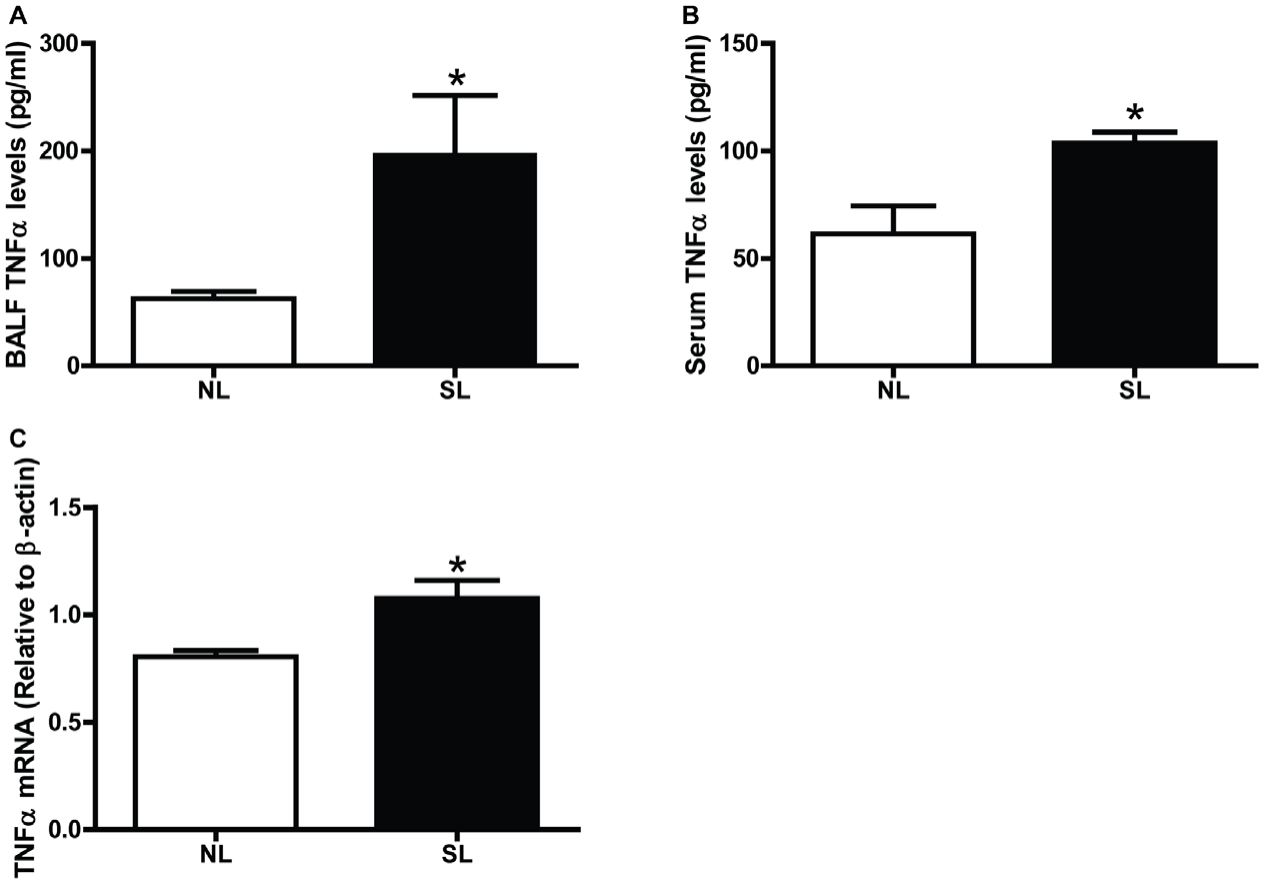
Neonatal overfeeding induces lung inflammatory cytokines on P150. The levels of TNF-α in BALF supernatant (A) and serum (B) were measured by Enzyme-Linked Immunoassays. The mRNA levels of TNF-α (C) in lungs were measured by quantitative real-time PCR. Data were expressed as mean± SEM, and the significant difference between two groups was analyzed by Student t-tests, *P<0.05.

The levels of TNF-α in serum and BALF supernatants were higher in the SL group than the NL group on P150 (Figure 5A and 5B). Comparing to the NL mice, the mRNA levels of TNF-α were significantly increased by 1.34-fold in the SL mice on P150 (Figure 5C).

### Neonatal Overfeeding Increases Lung Fibrosis

**Figure 6:**
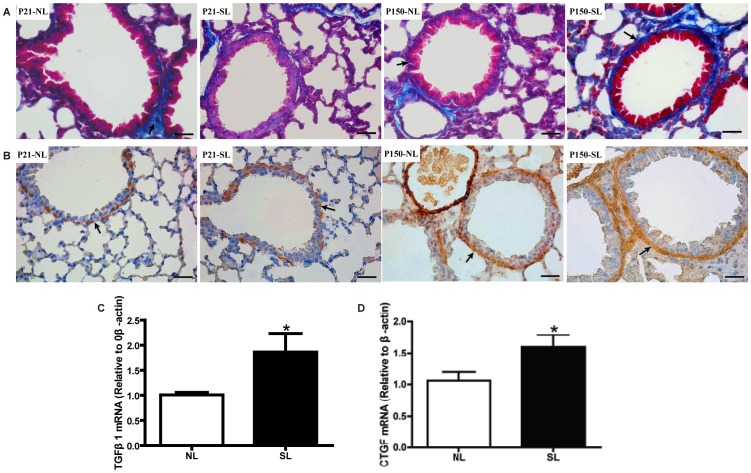
Neonatal overfeeding induces lung fibrosis on P150. The lungs were subjected to Masson staining (A) and α-SAM immunohistochemistry (B) for collagen in peri-bronchiolar areas on P21 and P150. Masson positive staining was blue and α-SAM positive staining was brown-reddish. The mRNA levels of TGF-β1 (C) and CTGF (D) in lungs were measured by quantitative real-time PCR. Data were expressed as mean± SEM, and the significant difference between two groups was analyzed by Student t-tests, *P<0.05.

To explore whether neonatal overfeeding induces lung fibrosis, Masson staining and α-SAM immunohistochemistry were performed to verify the collagen accumulation. Histology sections of connective tissue surrounding bronchial segments were shown in Figure 6. There was more fibrosis localized in peri-bronchiolar regions in the SL group compared with NL group on P150, colored by blue with Masson staining and brown-reddish with immunohistochemical staining for α-SAM (Figure 6A and 6B, right two panels). As shown in Figure 6A and 6B (left two panels), only few blue collagen and α-SAM positive cells were observed on P21 in both NL and SL mice. We could hardly find any difference in collagen accumulation around alveolar septum between the two groups at the two time points (data not shown). In addition, on P150, the mRNA levels of TGF-β1 (Figure 6C) and CTGF (Figure 6D) were significantly higher in SL group, suggesting neonatal overfeeding may be associated with lung fibrosis as adulthood.

## Discussion

In the current study, we observed that neonatal overfeeding induced by litter size reduction could enhance airway hyperresponsiveness (AHR) and lung inflammation, both of which were reported in other genetic types of obese mice, such as ob/ob, Cpefat mice and high fat diet induced obese mice [19]–[23], [30]. These studies suggest that airway hyperresponsiveness and inflammation are common features of obese mice. In addition, our data indicated that mice must maintain obesity for an extended period of time before the airway hyperresponsiveness was observed. Furthermore, our current study provided additional evidence that obesity resultant from neonatal overfeeding exhibited significant airway remodeling, characterized by collagen accumulation. To our knowledge, this is the first study to assess the relationship between neonatal overfeeding, airway inflammation and remodeling.

Rodent pups suckled in litters of varied sizes have been extensively used as experimental models in studying metabolic and behavior development. Taking them as experimental models, our present study confirmed previous findings and demonstrated that when ICR pups were raised in small litters (SL) with 3 pups per litter, they would show an accelerated weight gain prior to weaning in comparison with those raised in normal litters (NL) with 10 pups/litter. Even fed with the identical diet after weaning, SL animals maintained higher body weight throughout life. Additionally, significant glucose intolerance, and hyperleptinemia were observed in adult SL animals. These results are generally in accordance with earlier studies [27], [28], [31], [32].

The effects of obesity on pulmonary function have been extensively studied by Shore’s group with genetic and diet-induced obesity in mice [19]–[23]. However, the influence of obesity duration on respiratory system has rarely been investigated. In order to study this, we chose two time points: P21 and P150, and mice at these ages are generally equivalent to young and adult in human, respectively. In our neonatal overfeeding induced obesity model, AHR was only observed in adult mice (on P150). At obese state, lung volumes and expiratory flow rates were reduced, leading to closure of the smaller airways, which accounted for the apparent relationship between obesity and AHR. What surprised us is that airway responsiveness did not differ between neonatal overfeeding and control group on P21 though obvious difference in body weight has already existed. This could possibly be attributable to the fact that duration of obesity determines the development of AHR. These findings were supported by several other literatures. For instance, Cpefat mice had average body weight 23% and 84% more than controls at 7 and 14 weeks of age, whereas AHR was only found at 14 weeks [23]; and diet-induced obesity mice also displayed enhanced AHR in the older mice [21]. Furthermore, our study is consistent with some of the clinical observations that obese children younger than 5 years old did not show significant changes in pulmonary function [33]. All these findings suggest that extended duration of obesity is required to elicit subsequent AHR.

Airway inflammation is a critical factor contributing to AHR in the development of asthma [34]. In our study, more infiltrated inflammatory cells, especially macrophages as demonstrated by F4/80 immunohistochemistry, were observed in peri-bronchiolar areas and alveolar interstitium of neonatal overfeeding mice on P150; however, these changes were not found on P21. The same change was present in BALF cell counting, showing that total cells and classified cells (macrophages and lymphocytes) of BALF were significantly increased in neonatal overfeeding mice on P150. Though the classified cells of BALF in neonatal overfeeding mice on P21 were higher than their counterparts at the same period, the total cells of BALF were far fewer than those on P150. Therefore, our results suggest that neonatal overfeeding could induce macrophage recruitment, and these activated alveolar macrophages may increase pulmonary disease susceptibility [35]. Macrophage recruitment in the lungs of obese subjects may subsequently result in lymphocyte accumulation [36]. It is the reason why the lymphocytes increased followed by macrophages. However, our results were different from Lu’s reports [37], which showed that db/db mice exhibited AHR but BALF inflammatory cells were not significantly different from lean mice after air exposure. After challenged with ovalbumin, inflammatory cells from ob/ob mice were increased in the lung tissue to greater extent than wide-type mice, but the extent of increase in BALF was still lower than wild-type mice [20], [38]. One potential explanation for this disparity is the role of leptin, which could promote inflammatory cells in the lungs migrating into airway lumen (BALF). Ob/ob and db/db mice are genetically deficient in either leptin or leptin receptor, leading to the absence of anorexigenic and pro-inflammatory capacity of leptin [39]. In diet-induced obesity, as well as neonatal overfeeding mice, leptin is markedly increased [40], [41]. Leptin is a pro-inflammatory cytokine and is able to stimulate other inflammatory cytokine production from macrophages [42] and thereby enhances lung inflammation. In our present study, we found that TNF-α level of serum and BALF were increased on P150. TNF-α is inflammatory cytokine predominantly released from macrophages, which implicated in the chronic inflammatory status of both obesity and asthma [43]. Furthermore, exogenous administration of TNF-α was shown to enhance AHR [44], and TNFR2 signaling is required for the development of AHR in obese mice [45]. Taking together, these studies suggest that TNF-α released into the serum may circulate to the lungs and contribute to AHR.

Long-term low-graded airway inflammation may lead to airway remodeling [10], and both of these states are related to macrophage activation and overproduction of TNF-α [46], [47]. In our present study, obesity induced through neonatal overfeeding could promote collagen deposition around bronchi on P150. In addition, the transcriptional levels of CTGF and TGF-β1, which are important mediators of fibrosis and organ remodeling, were significantly upregulated in neonatal overfeeding mice on P150. Therefore, our data suggested that neonatal overfeeding induced obesity may be a potential risk for lung fibrosis, which is related to inflammatory cytokine (TNF-α and TGF-β1) released by increased macrophages.

In summary, our study suggests that neonatal overfeeding could increase pulmonary disease susceptibility by enhancing airway hyperresponsiveness and lung inflammation. It is plausible that the resulting airway hyperresponsiveness, lung inflammation and remodeling observed in these obese mice are the consequence of overproduction of inflammatory cytokines secreted from the active macrophages in the lung. Future studies will examine airway responsiveness after inflammatory stimuli and determine whether food restriction is sufficient to improve metabolic and respiratory phenotypes of these neonatal overfeeding mice.
